# Agronomic and hormonal approaches for enhancing flowering intensity in white Guinea yam (*Dioscorea rotundata* Poir.)

**DOI:** 10.3389/fpls.2023.1250771

**Published:** 2023-10-09

**Authors:** Jean M. Mondo, Paterne A. Agre, Géant B. Chuma, Robert Asiedu, Malachy O. Akoroda, Asrat Asfaw

**Affiliations:** ^1^ International Institute of Tropical Agriculture (IITA), Ibadan, Nigeria; ^2^ Institute of Life and Earth Sciences, Pan African University, University of Ibadan, Ibadan, Nigeria; ^3^ Department of Crop Production, Université Evangélique en Afrique (UEA), Bukavu, Democratic Republic of Congo; ^4^ Department of Agronomy, University of Ibadan, Ibadan, Nigeria

**Keywords:** *Dioscorea rotundata*, flowering intensity, agronomic and hormonal treatments, fruit set, tuber yield

## Abstract

Developing novel white Guinea yam (*Dioscorea rotundata*) varieties is constrained by the sparse, erratic, and irregular flowering behavior of most genotypes. We tested the effectiveness of nine agronomic and hormonal treatments to enhance flowering on *D. rotundata* under field conditions. Genotypes responded differently to flower-inducing treatments (*p*<0.001). Of the test treatments, pruning and silver thiosulfate (STS) were effective in increasing the number of spikes per plant and the flowering intensity on both sparse flowering and monoecious cultivars. STS and tuber removal treatments promoted female flowers on the monoecious variety while pruning and most treatments involving pruning favored male flowers. None of the treatments induced flowering on *Danacha*, a non-flowering yam landrace. Flower-enhancing treatments had no significant effect on flower fertility translated by the fruit set, since most treatments recorded fruit sets above the species’ average crossability rate. Flower-enhancing techniques significantly influenced number of tubers per plant (*p* = 0.024) and tuber dry matter content (DMC, *p* = 0.0018) but did not significantly affect plant tuber yield. Nevertheless, treatments that could enhance substantially flowering intensity, such as pruning and STS, reduced tuber yield. DMC had negative associations with all flowering-related traits. This study provided insights into white yam flower induction and suggests promising treatments that can be optimized and used routinely to increase flowering in yam crop, without significantly affecting flower fertility and tuber yield.

## 1 Introduction

Yam (*Dioscorea* spp.) is the fourth most important root and tuber crop worldwide and the second to cassava in West Africa (FAO, 2021). It plays significant food, economic, sociocultural, and religious importance in West Africa, where white yam (*D. rotundata*) is the most planted and consumed yam species (Obidiegwu et al., 2020). To unlock its full potential for food security and poverty alleviation, there is a need to develop novel yam varieties with high agronomic, adaptation, and tuber food quality (Darkwa et al., 2020; Agre et al., 2022). Developing such improved yam varieties is, however, hindered by sexual reproduction abnormalities following the crop domestication process that favored vegetative propagation using tubers at the expense of botanical seeds (Mondo et al., 2020; Cormier et al., 2021; Asfaw et al., 2022). Domestication alterations led to absent, low, erratic, or asynchronous flowering of most popular cultivars, high ovule abortion rates, and cross-compatibility barriers within and among yam species (Denadi et al., 2022; Mondo et al., 2022a). Besides, there is a disproportionately low female-to-male sex ratio (Mondo et al., 2020; Mondo et al., 2021a; Asfaw et al., 2022). Therefore, it is crucial to establish strategies to improve the yam sexual reproductive ability to ensure gene flow within and among yam species to achieve cross-breeding objectives.

Successful cases of improved flowering ability and intensity for root and tuber crops have been achieved using agronomic and hormonal approaches (Lebot, 2010; Muthoni et al., 2012; Ceballos et al., 2017; Hyde et al., 2020; Luthra et al., 2020; Pineda et al., 2020a; Pineda et al., 2020b; Oluwasanya et al., 2021; Hyde and Setter, 2022). The most effective treatments included grafting, pruning, proper soil fertility management, choice of most suitable sites, treatment with plant growth regulators (PGR), and photoperiod and temperature manipulation (Mondo et al., 2020). For instance, silver thiosulfate (STS), an anti-ethylene PGR, and the synthetic cytokinin (benzyladenine BA) have been successfully used in cassava to induce profuse flowering and increase female flower numbers and fruits, respectively, under controlled and field conditions (Hyde et al., 2020; Pineda et al., 2020a; Oluwasanya et al., 2021). Promising results on potato flowering intensity were achieved with weekly sprays of gibberellic acid (GA_3_), an endogenous hormone (Luthra et al., 2020). Agronomic manipulations such as pruning of branches and tuber removal have been proven effective in inducing profuse and long flowering on cassava and potato, respectively, under both field and greenhouse conditions (Luthra et al., 2020; Pineda et al., 2020a; Oluwasanya et al., 2021). Recently, it has been found that maintaining long-day photoperiod and cool temperatures could induce flowering in cassava (Hyde and Setter, 2022). Combining treatments is encouraged to exploit the synergistic effect on flower induction, duration, and feminization (Pineda et al., 2020a; Oluwasanya et al., 2021). However, routine application of such practices to improve flowering efficiency in white yam is still limited (Mondo et al., 2020). We hypothesized that their use in breeding programs, after a series of testing for efficiency, could ease reproductive constraints hindering white yam breeding.

This study, therefore, assessed: (1) the effectiveness of agronomic (pruning and tuber removal) and hormonal (STS, BA, and GA_3_) treatments, singly and combined, in improving flower production and fruit set in field conditions; and (2) the influence of these flower-enhancing treatments on agronomic performance of test yam clones.

## 2 Materials and methods

### 2.1 Study site

This study was conducted from April 2021 to March 2022 at the International Institute of Tropical Agriculture (IITA), Ibadan, Nigeria (7°29′ N and 3°54′ E). The yam field was established in April 2021, and the flowering occurred from early August to mid-October 2021. Tuber and fruit harvesting were done in January 2022. The soil of the experimental site is of Alfisol type, acidic (pH = 5.5), and sandy loam, with low organic carbon (OC) and mineral contents. The effective cation exchange capacity (CEC), the total amount of exchangeable cations (sodium, potassium, calcium, and magnesium), is low for the proper release of nutrients to the soil solution (**Supplementary Table 1**, **Supplementary Figure 1**).

Weather parameters during the flowering window period are presented in **Supplementary Figure 2**. Among weather conditions, solar radiation fluctuated much across flowering weeks (107.3 MJ m^−2^ day^−1^ in week 6 to 188.6 MJ m^−2^ day^−1^ in week 21). The wind speed varied with weeks (week 15 had the lowest wind speed: 0.5 km ha^-1^ while week 4 had the highest wind speed: 5.1 km ha^-1^). Maximum and minimum temperatures followed similar trends across weeks: week 21 had the highest minimum (27.5°C) and maximum temperatures (28.6°C). The relative humidity ranged from 72.0 to 96.1%. The total rainfall was 920.3 mm during the flowering window (**Supplementary Figure 2**).

### 2.2 Plant materials, field management, and treatment application

To test the flower-enhancing treatments, three clones of different flowering intensities were selected: non-flowering (*Danacha*), sparse flowering (TDr8902665), and monoecious (TDr1669012), while one profuse flowering clone (TDr1100873) was used as satellite plot to monitor the flowering in the season. These genotypes’ flowering behavior was identified based on IITA historical flowering data information from 2010 to 2020. All these genotypes were diploids (Mondo et al., 2022a). Field management followed the standard recommendations for the yam crop (Mondo et al., 2022b). Larger spacing (2 m between rows and 1.5 m within rows) was used to facilitate treatment and data collection. Individual plants were staked using 2.5 m long bamboo poles. No fertilizer or supplemental irrigation was applied. Fields were kept free of weeds with regular manual weeding.

Different agronomic and hormonal treatments were applied as follows:

(1) Silver thiosulfate (STS) spraying started 14 days before flowering and extended to a post-flower appearance. The 0.02 M STS was prepared by slowly pouring 1 part of 0.1M silver nitrate (1 g L^-1^ AgNO_3_) into four parts of 0.1M sodium thiosulfate (4 g L^-1^ Na_2_S_2_O_3_), yielding a 20-mM stock solution (Hyde et al., 2020; Oluwasanya et al., 2021). Before spraying, the stock solution was diluted with distilled water to the appropriate concentration. Notably, a preliminary dose test was conducted to prevent hormone phytotoxicity on yam leaves. The control treatment consisted of applying distilled water with a few drops (3 cc per liter) of 0.1 Tween 20^®^ (ethoxylated sorbitan monolaurate (nonionic surfactant)).
(2) Gibberellic acid (GA_3_) treatment consisted of weekly foliar sprays on adult plants that had already flowered at least once. The solution was made of 50 ppm (50 mg/L) of GA_3_ in distilled water with a few drops of 0.1 Tween 20^®^, as described by Luthra et al. (2020). The control plots were sprayed with distilled water at the same frequency. The leaves were sprayed thoroughly until they became fully wet.
(3) Pruning was performed periodically, starting soon after the first flowering events, as directed by Pineda et al. (2020a). It consisted of reducing excess leaves, lateral branches, and the shoot apical region using sharp scissors.
(4) Benzyladenine (BA) foliar spraying was initiated eight weeks after planting when singly applied, or after pruning when in combination (Pineda et al., 2020a). BA was applied weekly until the transition from flowers to fruits was observed. A 50 ppm (w/v) BA solution was prepared mixing active compounds with distilled water and 0.1 Tween 20. When in combination, the solution was sprayed on plants immediately after pruning.
(5) Tuber removal was conducted to discourage tuber formation and was repeatedly done every 15 days, starting from flower initiation.

As suggested by Oluwasanya et al. (2021), combining treatments has synergistic effects on flower induction and feminization. The above-described treatments were combined as in **Table 1**.

**Table 1:** Proposed treatment combinations.

Treatment code	Treatments*
T0	Control
T1	STS
T2	GA_3_
T3	Pruning (P)
T4	BA
T5	P + BA
T6	Tuber removal
T7	P+STS
T8	P+STS+BA

*These combinations were sourced from the literature on root and tuber crops’ flower-enhancing treatments. STS, Silver thiosulfate; BA, Benzyladenine; GA_3_, Gibberelic acid and P, pruning.

Regardless of the treatment, foliar spraying was done in the morning hours, when the relative humidity was still high and the evapotranspiration was low, to favor the product uptake. Besides, since yam leaves are very leathery, a few drops (3 cc per liter) of 0.1 Tween 20 were used (as described above) as a surfactant and adjuvant to facilitate and accentuate the emulsifying, dispersing, spreading, wetting of the chemicals sprayed on the leaves, to improve their penetration and efficiency, and thus preventing showers to wash away the solutions before acting. All the treatments along with the three genotypes were arranged in a split–plot design with two study factors: the genotype (as the main plot) and the flower induction practice (as subplots). An experimental unit (subplot) was made of five plants, making a total of 405 plants (5 plants per subplot × 9 treatments × 3 genotypes × 3 replications) for the entire experiment (**Supplementary Figure 3**). Three satellite plots (with 15 plants each) were randomly included in each replication.

### 2.3 Data collection and statistical analyses

Data on parameters described in **Supplementary Table 2** were recorded following the yam crop ontology (Asfaw, 2016) using FieldBook App (Rife and Poland, 2014). During the flowering period, plots were monitored daily to determine the flower initiation time. Once flowers were initiated and properly formed (two weeks after the first flowering event), the number of spikes and flowers per spike were counted. Male and female flowers were counted separately on monoecious plants. Female flowers from each treatment were then assessed for fertility using the *in vivo* testing method as described by Mondo et al. (2020; 2021b). Hand pollination (consisting of removing the anther from the male parent’s flower and depositing it on the stigma of the female parent’s flower) was conducted five days after female flowers were bagged (Mondo et al., 2022b). On these plots, hand pollination was continuously performed on flowering genotypes and treatments until the end of the flowering window. Therefore, an unequal number of female flowers were pollinated per treatment and genotype since clones had different flowering intensities and windows, and they responded differently to flower enhancing treatments. A set of three male parents ready at the experiment period were used as pollen sources regardless of the female genotypes. These included TDr1614005, TDr1621010, and TDr1613701. Corresponding fruit and seed sets from these cross-combinations were recorded. The flowering and fruiting rates for a genotype or a treatment were calculated as the ratio between the number of plants that have effectively flowered or fruited over the total number of plants in a plot that received that genotype or treatment. Plants were left in the field until the senescence period, and the effects of flower-enhancing treatments on tuber yield, number of tubers per plant, and dry matter content were assessed.

Data were then entered into Microsoft Excel 2016, a database was created, and descriptive statistics analysis was performed on all parameters (flowering and yield related) to allow assessing data anomalies. It consisted of calculating both central and dispersion tendencies, such as the mean, the standard deviation (SD), and the coefficient of variation (CV). The maximum and minimum values were determined at the same time. The CV enabled identifying highly dispersed variables that required transformation (followed by visualizing the shape of variable histogram). Thus, the Kolmogorov-Smirnov normality test was performed for all variables with high CV. The test involved analyzing the *p*-value and the probability diagram to see whether the data points closely followed the adjusted distribution’s right side (Gupta, 2021). Arctangent (variable)^2^ was used to convert the data; new variables obtained through transformations were exported into Minitab 21 (Gupta, 2021) for analysis. Treatments and varieties effects on yam flowering and yield parameters were assessed using the analysis of variance (ANOVA) at a 5% probability threshold following the split-plot design. Flower-enhancing treatment was regarded as the primary factor, and the variety as the secondary factor. Mean values were separated using the least significant difference of means (LSD) post-ANOVA test. The histograms were plotted with a confidence interval (CI) = 95%. All analyses were conducted using different packages implemented in R. It is noteworthy that plots where tuber removal was applied were not considered when assessing the influence of flower-enhancing treatments on tuber yield, number of tubers per plant, and DMC.

## 3 Results

### 3.1 Summary descriptive statistics

Regardless of the treatment, the landrace *Danacha* did not flower at all. TDr1669012 had 83.3% flowering and 46.4% fruiting rates while TDr8902665 was characterized by 28.2% flowering and 10.9% fruiting rates. The satellite line, TDr1100875, was highly profuse and fertile (95.2%) (**Supplementary Table 3**). Based on flower induction treatments, all TDr1669012 plants subjected to pruning combined with the BA (P+BA) flowered. The lowest flowering rate was on the control with no treatment (60%) for this genotype. For TDr8902665, the highest flowering rate was on the STS (58.3%) and the lowest was on BA (14.3%). The trend was similar for fruiting, with the highest fruiting rates being on P+BA and tuber removal (60%) for TDr1669012 and the lowest rates on P+STS and pruning (**Table 2**). Five of the nine treatments allowed fruiting on TDr8902665 (P+BA+STS, P+STS, pruning, STS, and tuber removal), the highest rate being on plots treated with STS (33.3%). The trend was the same across weeks though the highest values were observed about two weeks after the first flowering event and then started decreasing.

**Table 2:** Effects of flower induction techniques on white yam fertility.

Treatment	Flowering rate (%)				Fruiting rate (%)			
	Danacha	TDr1669012	TDr8902665	Mean	Danacha	TDr1669012	TDr8902665	Mean
BA	0.0	66.6	14.3	27.0	0.0	44.4	0.0	14.8
Control	0.0	60.0	23.1	27.7	0.0	40.0	0.0	13.3
GA_3_	0.0	88.9	41.7	43.5	0.0	55.6	0.0	18.5
P+BA	0.0	100.0	42.9	47.6	0.0	60.0	0.0	20.0
P+BA+STS	0.0	80.0	50.0	43.3	0.0	40.0	16.7	18.9
P+STS	0.0	66.6	18.2	28.3	0.0	33.3	9.1	14.1
Pruning	0.0	88.9	33.3	40.7	0.0	33.3	25.0	19.4
STS	0.0	80.0	58.3	46.1	0.0	50.0	33.3	27.8
Tuber removal	0.0	80.0	30.0	36.7	0.0	60.0	20.0	26.7
Mean	0.0	79.0	34.6	37.9	0.0	46.3	11.6	19.3
SD	0.0	12.8	14.8	8.3	0.0	10.6	12.7	4.8

STS, Silver thiosulfate; BA, Benzyladenine; GA_3_, Gibberelic acid; P, pruning; SD, standard deviation.

Results presented below are those collected from the two flowering test genotypes at two weeks (within the flowering window) when the flowering was optimal.

### 3.2 Influence of flower induction treatments on flowering traits

The number of spikes per plant varied significantly with the genotype (*p*<0.001), the flower induction treatments (*p*<0.001), and the genotype × treatment interactions (*p*=0.002). TDr1669012 had consistently higher numbers of spikes per plant (61) than TDr8902665 (6), regardless of the treatment (**Table 3**). Among treatments, pruned plants had the highest number of spikes on TDr1669012 (140), while the highest number of spikes for TDr8902665 was on STS (13). The lowest numbers of spikes were on the control plots for both genotypes. Concerning the total number of female flowers per plant, only the treatment had a significant influence on the outcome (*p*=0.023), with better results being achieved on tuber removal for TDr1669012 (269) and STS (149) for TDr8902665 (**Table 3**). Other treatments with good results were P+BA+STS, BA and GA_3_ for TDr1669012 and pruning for TDr8902665. Both genotypes (*p*<0.001) and treatments (*p*=0.0137) significantly influenced the total number of flowers per plant. The monoecious clone TDr1669012 had a substantially higher number of flowers (1067) than TDr8902665 (64). Pruning had the best results for TDr1669012 (2446 flowers), while STS (149 flowers) and pruning (114 flowers) had better results on TDr8902665 (**Table 3**).

**Table 3:** Influence of flower induction treatments on flowering traits of two yam varieties.

Genotype	Treatment	Number of spikes per plant	Number of female flowers per plant	Total number of flowers per plant
TDr8902665	BA	4.5 ± 0.4^e^	40.5 ± 2.9^d^	40.5 ± 2.9^b^
	Control	2.0 ± 0.0^f^	25.0 ± 0.0^e^	25.0 ± 0.0^c^
	GA_3_	7.2 ± 1.0^e^	74.1 ± 21.4^c^	74.1 ± 21.4^a^
	P+BA	3.1 ± 0.8^f^	38.0 ± 21.8^d^	38.0 ± 21.8^b^
	P+BA+STS	3.5 ± 1.5^f^	37.7 ± 18.0^d^	37.7 ± 18.6^b^
	P+STS	3.0 ± 0.0^f^	33.0 ± 0.0^e^	33.0 ± 0.0^c^
	Pruning (P)	11.7 ± 5.8^d^	114.1 ± 60.0^b^	114.1 ± 60.0^a^
	STS	12.9 ± 1.6^c^	149.2 ± 9.0^b^	149.2 ± 9.0^a^
	Tuber removal	4.7 ± 2.4^e^	61.7 ± 49.8^c^	61.7 ± 49.8^a^
Mean ± SD		5.8 ± 4.4B	63.7 ± 49.1A	63.7 ± 49.1B
TDr1669012	BA	22.7 ± 7.6^c^	211.7 ± 130.8^ab^	428.3 ± 147.1^b^
	Control	15.5 ± 0.4^c^	28.5 ± 0.4^f^	228.6 ± 1.1^c^
	GA_3_	32.3 ± 17.9^b^	175.5 ± 129.0^b^	429.3 ± 255.7^b^
	P+BA	66.5 ± 47.8^ab^	31.5 ± 17.6^e^	1227.8 ± 946.9^a^
	P+BA+STS	40.5 ± 14.3^b^	211.4 ± 78.9^ab^	517.1 ± 304.0^b^
	P+STS	99.5 ± 74.7^a^	69.0 ± 9.8^c^	1806.5 ± 1370^a^
	Pruning (P)	139.5 ± 82.4^a^	99.0 ± 28.6^c^	2446.0 ± 1591^a^
	STS	76.5 ± 0.4^ab^	77.7 ± 0.6^c^	1243.0 ± 35.1^a^
	Tuber removal	84.3 ± 50.7^a^	269.2 ± 141.8^a^	1274.7 ± 888.0^a^
Mean ± SD		64.1 ± 58.7A	130.4 ± 117.1A	1066.8 ± 998.7A
Grand Mean ± SD		35.0 ± 30.8	97.0 ± 95.8	565.2 ± 318.8
CV (Block×Genotype)		7.36	1.87	1.03
CV (Block×Treatment×Genotype)		7.77	2.51	1.32
*P*-value	Genotype	<0.001^**^	0.250^ns^	<0.001^***^
	Treatment	<0.001^***^	0.023^*^	0.0137^*^
	Treatment×Genotype	0.002^**^	0.469^ns^	0.218^ns^

STS, Silver thiosulfate; BA, Benzyladenine; GA_3_, Gibberelic acid; P, pruning; SD, standard deviation. ^ns^, *, **, *** not significant, significant, and highly significant at 5% p-value threshold of LSD test. Uppercase letters (A and B) refer to separation according to the main factor (genotype), while lowercase letters are for the treatments or their interactions. ANOVA was performed after Arctan(x)^2^ transformation for data with coefficient of variation (C.V.)>15%.

**Figure 1:**
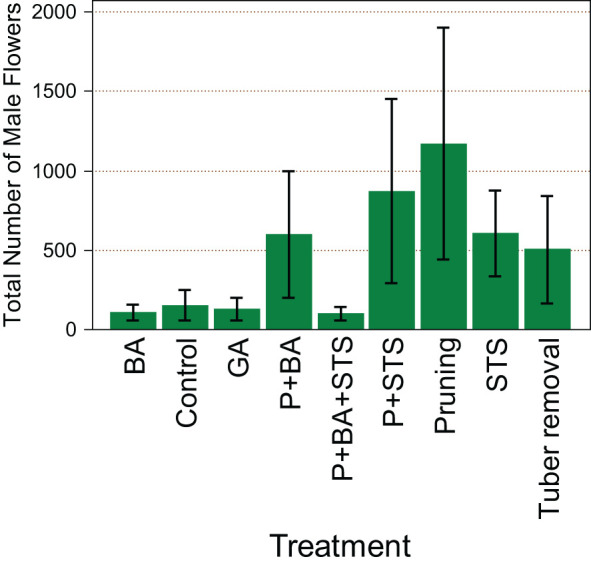
Influence of flower induction treatments on the number of male flowers on the monoecious *D. rotundata* genotype TDr1669012. STS, Silver thiosulfate; BA, Benzyladenine; GA_3_, Gibberelic acid; P, pruning.

Treatments had a significant effect (*p*<0.001) on the number of male flowers from the monoecious genotype TDr1669012. Pruning (1809) seems to favor more male flowers than any other treatment (**Figure 1**). The other treatment with the highest number of male flowers was P+STS (1059), while P+BA+STS had the least number of male flowers (200).

### 3.3 Influence of the flower induction practices on fruit set

**Figure 2:**
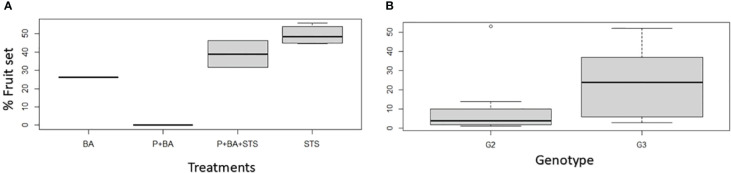
Influence of flower induction treatments on hand pollination success rates in *D. rotundata*: **(A)** treatments’ effect, **(B)** genotypic effect. STS, Silver thiosulfate; BA, Benzyladenine; P, pruning; G2, TDr8902665; G3, TDr1669012.

For treatments on which hand pollination was conducted, we realized that the flower fertility was not affected much by flower induction treatments since most treatments (26.3 – 51.8%) exceeded the crop average crossability rate, which is 23% for *D. rotundata* at IITA Nigeria (**Figure 2**). Natural pollination results from treated plants are presented in **Supplementary Figure 4**.

### 3.4 Agronomic performance of three white yam varieties under flower induction management

Agronomic traits significantly varied with genotypes, treatments (except the tuber yield), and the interactions genotypes × treatments (**Table 4**). The highest number of tubers per plant (3) was recorded on control plots and P+STS for TDr8902665 and P+BA for TDr1669012. The highest yields were from untreated control plots (48.8 t ha^-1^), P+BA+STS (48.2 t ha^-1^), and GA_3_ (46.7 t ha^-1^) plots all on TDr1669012 while the lowest yield was from *Danacha* treated with P+BA (13.8 t ha^-1^). Overall, the highest dry matter content (DMC) was on GA_3_ and P+BA+STS (40%) while the lowest DMC was on pruning alone (27%).

**Table 4:** Influence of flower induction treatments on tuber yield-related traits of three yam varieties.

Genotype	Treatment	Number of tubers per plant	Tuber yield (t ha^-1^)	DMC (%)
Danacha	BA	2.0 ± 0.0^ab^	34.4 ± 0.1^b^	38.7 ± 0.1^ab^
	Control	1.0 ± 0.0^c^	20.8 ± 0.8^bc^	33.7 ± 0.8^cd^
	GA_3_	1.0 ± 0.0^c^	16.7 ± 1.5^c^	35.1 ± 1.7^c^
	P+BA	1.0 ± 0.0^c^	13.8 ± 2.2c	34.3 ± 1.4^cd^
	P+BA+STS	1.7 ± 0.6^c^	19.2 ± 8.0^bc^	35.3 ± 3.6^c^
	P+STS	1.0 ± 0.0^c^	17.8 ± 0.1^c^	35.3 ± 0.1^c^
	Pruning (P)	1.0 ± 0.0^c^	16.4 ± 6.5^c^	34.0 ± 0.5^cd^
	STS	1.3 ± 0.6^c^	22.1 ± 3.0^bc^	33.1 ± 2.5^cd^
Mean ± SD		1.3 ± 0.5B	19.3 ± 7.0B	34.5 ± 2.6A
TDr8902665	BA	1.3 ± 0.6^c^	35.8 ± 4.9^b^	35.3 ± 1.6^c^
	Control	2.7 ± 1.5^a^	39.6 ± 22.9^ab^	37.8 ± 0.6^b^
	GA_3_	1.0 ± 0.5^c^	42.3 ± 0.1^ab^	40.0 ± 0.1^a^
	P+BA	2.0 ± 1.0^ab^	29.1 ± 5.9^bc^	37.8 ± 0.8^b^
	P+BA+STS	1.7 ± 1.2^c^	21.2 ± 11.5^c^	40.4 ± 2.2^a^
	P+STS	2.7 ± 1.2^a^	34.4 ± 8.0^b^	37.7 ± 2.1^b^
	Pruning (P)	2.0 ± 0.5^ab^	42.2 ± 0.1^ab^	30.0 ± 0.1^d^
	STS	1.0 ± 1.0^c^	19.0 ± 5.3^c^	34.2 ± 3.3^cd^
Mean ± SD		1.8 ± 0.5B	35.1 ± 12.9A	35.9 ± 4.0A
TDr1669012	BA	2.0 ± 0.6^ab^	35.4 ± 15.2^b^	33.6 ± 3.7^cd^
	Control	1.4 ± 1.5^c^	48.8 ± 6.9^a^	30.3 ± 0.3^d^
	GA_3_	2.3 ± 0.0^ab^	46.7 ± 6.6^a^	28.7 ± 1.6^e^
	P+BA	2.7 ± 1.0^a^	33.7 ± 4.0^b^	31.1 ± 1.7^d^
	P+BA+STS	2.3 ± 1.2^ab^	48.2 ± 29.5^a^	31.8 ± 1.7^d^
	P+STS	2.3 ± 1.2^ab^	41.2 ± 19.6^ab^	30.7 ± 0.5^d^
	Pruning (P)	1.7 ± 0.0^b^	43.2 ± 19.4ab	27.3 ± 1.6^e^
	STS	2.3 ± 0.0^ab^	35.0 ± 24.1^b^	30.9 ± 4.1^d^
Mean ± SD		2.0 ± 0.9A	40.0 ± 15.8A	30.5 ± 2.8B
Grand Mean ± SD		1.7 ± 1.0	31.4 ± 15.2	33.6 ± 3.9
CV (Block×Genotype)		1.49	13.67	0.31
CV (Block×Treatment×Genotype)		1.67	15.83	0.25
*P*-value: Treatment		0.0241^*^	0.838^ns^	0.0018^**^
Genotype		<0.001^***^	<0.001^***^	<0.001^***^
Treatment×Genotype		0.0021^**^	0.0018^**^	0.0054^**^

STS, Silver thiosulfate; BA, Benzyladenine; GA_3_, Gibberelic acid; P, pruning; SD, standard deviation. ^ns^, *, **, *** not significant, significant, highly and very highly significant at 5% p-value threshold of LSD test. DMC, dry matter content. ANOVA was performed after Arctan(x)^2^ transformation of data with a coefficient of variation (C.V.)>15%. Uppercases (A and B) refer to separation according to the main factor (genotype), while lowercase letters (a, b, c) are for the treatments or their interactions.

### 3.5 Correlation coefficients between agronomic parameters and flowering traits

**Figure 3:**
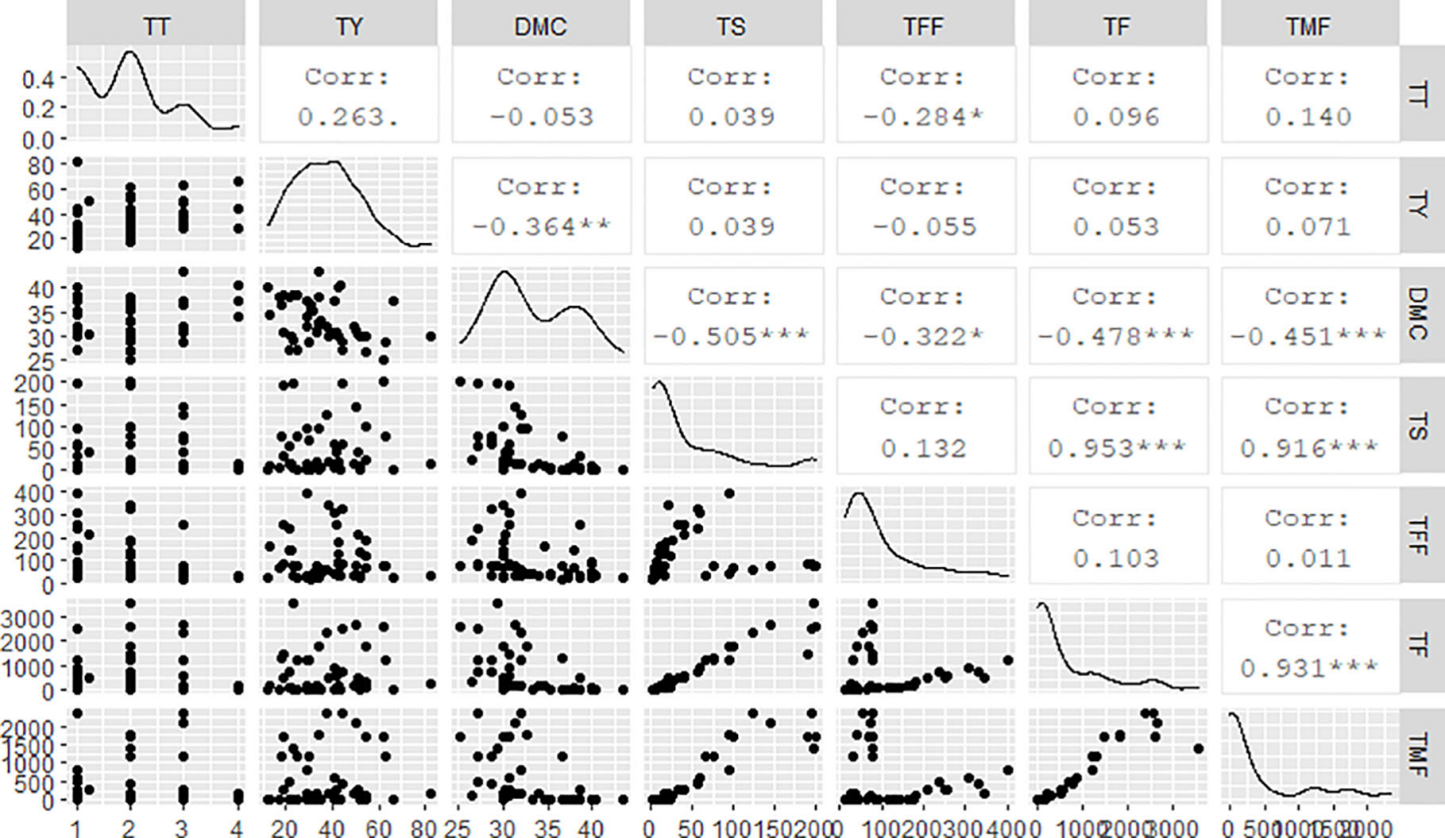
Regression analyses among flowering and tuber yield traits. TT, total number of tubers per plant; TY, tuber yield; DMC, dry matter content; TS, total number of spikes per plant; TFF, total number of female flowers; TF, total number of flowers per plant; TMF, total number of male flowers.

Results from this study showed negative associations between the tuber DMC and all flowering-related traits (**Figure 3**): total number of spikes (r = -0.50***), total number of female flowers (r = -0.32**), total number of flowers (r = -0.48***), and total number of male flowers (r = -0.45**). Besides, DMC had a negative correlation with tuber yield (r = -0.36***). Tuber yield was positively associated with number of tubers per plant (r = 0.378**). On the other hand, total number of tubers per plant was negatively correlated with total number of female flowers (r = -0.284*). Generally, there were strong positive correlations among flowering-related traits: number of spikes per plant was significantly and positively associated with total number of flowers per plant (r = 0.95***) and total number of male flowers per plant (r = 0.91***). A strong positive correlation (r = 0.93***) existed as well between total number of female flowers and total number of flowers per plant.

## 4 Discussion

### 4.1 Influence of agronomic and hormonal treatments on flowering intensity in yam

Yam is characterized by low flowering rates (low prolificacy), especially for female genotypes. Through agronomic and hormonal treatments, this study tried to improve the number of female flowers on sparse flowering female and monoecious genotypes. Pruning and STS were effective in increasing the number of spikes per plant and the floral intensity on both sparse flowering and monoecious cultivars. The STS and tuber removal treatments promoted female flowers on the monoecious variety while pruning and most treatments involving pruning favored male flowers. Results showed positive effects of STS and pruning on boosting flowering under field conditions, as Oluwasanya et al. (2021) reported on cassava, another starchy tuber crop. Such finding suggests that these promising treatments could be exploited to substantially ease the low flowering intensity challenges hindering yam breeding efforts. Oluwasanya et al. (2021) noted that combining pruning and STS treatments led to an additive increase in flower abundance on cassava sparse flowering genotypes. Such a positive effect of the anti-ethylene treatment STS on flowering was also reported by Hyde et al. (2020) on cassava under greenhouse conditions. These authors argued that the STS prolongs the flower bud formation such that there is an increase in the number of flowers formed. They showed that poor flowering ability in crops like cassava is partly linked with the ethylene inhibition of inflorescence development and flower formation. Oluwasanya et al. (2021) reported that applying the STS alone increased the total number of flowers by over two-fold relative to the no-STS controls. Transcriptomic analysis of gene expression in tissues of the apical region and developing inflorescence in cassava revealed that flower-enhancing treatments with pruning and STS create widespread changes in the network of hormone signaling and regulatory factors beyond ethylene and cytokinin (Oluwasanya et al., 2021). Though Amanze (2017) reported that a high level of Gibberellic acid (GA_3_) is capable of inducing flowering, fruiting and seed production potential in flowering and non-flowering yam varieties; none of the treatments induced flowering in *Danacha*, a non-flowering landrace. This inefficiency on *Danacha* indicates the need for more search for a flower inducer in such yam genotypes. Treatments such as photoperiod and temperature manipulations should be tested in future studies to assess whether they could induce flowering on such non-flowering yam genotypes, as they did on cassava, another root crop (Hyde and Setter, 2022). Besides, we recommend yam breeders to try out broad spectrum flower inducers, flower enhancers, and fruit retainers that proved efficient in different crops, including vegetables, fruits, legumes, and flowers. These are commercial products on the market with different trade names (e.g. AGRLGOLD, AGRIGOLD, AGRI-GOLD) depending on the region or country you are in. In addition to trying new products, yam breeders should consider optimizing product doses and application practices of promising treatments to assess whether they could break *Danacha* non-flowering behavior. Such product dose and application methods’ optimization should particularly seek at increasing efficiency, minimizing phytotoxicity and product wastage, as well as the amount of residues introduced into plant debris and soil.

### 4.2 Influence of agronomic and hormonal treatments on sex ratio in monoecious plants

There were significant genotypic and treatment effects on the number of female and male flowers on monoecious plants. Regardless of genotype, STS and tuber removal treatments promoted female flowers, while pruning and most treatments involving pruning favored male flowers. These results contrasted findings in cassava that pruning and STS only increase flower numbers while having minimal influence on sex ratios (Oluwasanya et al., 2021). These authors showed that pruning and STS treatment proportionally increased the number of female and male flowers, such that female-to-male ratios were relatively unchanged. The positive effects of pruning on enhancing the flowering of tuber crops (such as cassava) were also reported by Pineda et al. (2020a). From our study, pruning, P+BA, P+STS and tuber removal favored the production of more males than other treatments. Since the equilibrium sex ratio of 1:1 expected from the Fisherian theory is seldom achieved in *Dioscorea* species, the latter being characterized by a significant male bias (Mondo et al., 2020), findings from this study are critical for yam breeders to favor the right balance of male (pollen) and female gametes (ovules). For instance, yam breeders are advised to use STS when more female flowers are desired, while they should adopt pruning when male flowers are needed to favor the population outcome in a breeding scheme.

Gibberelic acid (GA_3_) is a reputed flower inducer in several crops (Hassankhah et al., 2018) but was not much effective in yam as we expected, its outcome being significantly lower than the best performing treatments (STS and pruning). In contrast to our findings, several reports showed that when applied at the right concentration, the GA_3_ alters the flowering pattern of plants (Hassankhah et al., 2018). These authors showed that GA_3_ application significantly increases the number of male flowers, total flowers, and male–to–female flower ratio per branch. The inefficiency of GA_3_ on yam can be attributed either to suboptimal concentration or to the crop non-responsiveness. For instance, previous reports showed that the effect of GA_3_ on flowering depends on plant species and product concentration. In biennial and long-day plants, GA_3_ promotes flowering, whereas in other plants, including hermaphrodite trees, it inhibits flowering and increases biennial bearing (Wilkie et al., 2008; Goldberg-Moeller et al., 2013). The role of GA_3_ in flower induction of monoecious and dioecious plants is different and more complex; GA_3_ in some monoecious plants increases the number of male flowers, e.g. cucumber (Iwahori et al., 1970) and in some others it increases the female flowers, e.g. maize (Young et al., 2004). Based on molecular evidence, GA_3_ regulates the development of flowers through activating some genes such as *LEAFY* (LFY) and *AP1* (Jack, 2004). Other genes, like *UFO*, *WUS*, and *SEP3* can act as co-factors for *LFY* in the activation of genes that specify the identity of the flower organ (Rijpkema et al., 2010; Murai, 2013). Transcript profiling of cassava tissues in the inflorescence region indicated that flower-enhancing treatments affect signaling components in multiple hormones and flowering regulatory pathways, including those involving auxin, GA_3_, ethylene, jasmonate, and ABA (Oluwasanya et al., 2021). A similar transcriptomic study should be conducted on yam to understand more deeply how agronomic and hormonal treatments regulate flowering outcomes.

### 4.3 Influence of agronomic and hormonal treatments on flower fertility and fruit set

In addition to its effects on flowering, STS increased fruit numbers such that the fraction of female flowers that set fruit increased in treatments that involved STS (**Table 2**, **Figure 2**). In contrast to what we expected, flower fertility was not affected much by floral induction treatments, since the hand pollination success rates for most treatments (26.3 – 51.8%) exceeded the crop average crossability rate (23%) at IITA, Nigeria (Mondo et al., 2022a). Hand pollination favored higher fruit set than natural pollination by insects (**Supplementary Figure 4**). Pollination success ranged from 26.3 to 51.8% on hand-pollinated flowers, while it ranged from 1.5 to 37% for open (natural) pollination. The insects’ inefficiency was previously listed as a major factor of low natural pollination success in yam (Akoroda, 1985; Segnou et al., 1992; Mondo et al., 2022b). This insect’s inefficiency is associated with a low visitation rate, limited movements and selectivity (Martin et al., 1963; Mondo et al., 2020). Hand pollination is, therefore, used as an alternative solution; it is 2–3 times more efficient than natural pollination by insects (Akoroda, 1983; Segnou et al., 1992). While ethylene is widely recognized as playing a role in fruit ripening (Pech et al., 2012), it also affects the early stages of fruit development and fruit set. In peas (*Pisum sativum*) and Arabidopsis, failure to develop fruit in the absence of pollination has been associated with ovary senescence arising from increased ethylene biosynthesis in ovaries (Orzáez and Granell, 1997; Carbonell-Bejerano et al., 2011). In Zucchini squash (*Cucurbita pepo*), blocking ethylene perception by STS extended ovule lifespan and increased the chance of developing fruit either by pollination or by parthenocarpy in response to gibberellins (Martínez et al., 2013). In addition to increasing flowering intensity in yam, some treatments such as STS had positive outcome on fruiting rate. Such finding opens an avenue for devising strategies that could break low cross pollination success that hinders yam breeding efforts. For instance, yam pollination success is estimated at 23 and 31% for *D. rotundata* and *D. alata*, respectively (Mondo et al., 2022a), a situation that slows generation of genetically variable offspring for selection.

### 4.4 Influence of agronomic and hormonal treatments on tuber yield and other tuber-related traits

We found that flower-enhancing treatments significantly affected number of tubers per plant and tuber dry matter content but had no significant effect on tuber yield. Treatments favoring profuse flowering reduced agronomic performance, especially DMC. Several past studies support the good yield performance recorded on plants treated with GA_3_. They showed that GA_3_ can stimulate the expansion and production of new tubers, increasing tuber weight and yield (Kim et al., 2005; Yoshida et al., 2008; Gong et al., 2016). Zhou et al. (2021) showed that GA_3_ is mainly involved in tuber growth at the early expansion stage since it acts on cell expansion and division during tissue development. On cassava, Oluwasanya (2020) noted an improvement in tuber yields with flower-enhancing treatments (STS and BA) compared to untreated controls. Regulation benefits on tuber and storage root development of phytohormones used in the present study (gibberellin, cytokinin, and benzylaminopurine) were also reported for other root and tuber crops (Li et al., 2020; Kondhare et al., 2021; Zierer et al., 2021). The results did not confirm our assumption that stimulating abundant flowering could negatively affect tuber bulking since no significant effect was demonstrated among treated plots and untreated ones. However, there was negative correlation between DMC and all flowering-related traits. This negative association could be related to the photosynthetic competition between many yam flowers and fruits induced on treated plants that represented large sinks compared to the primary sinks, storage roots and vegetative shoots (Hyde et al., 2020; Oluwasanya, 2020).

## 5 Conclusions

Agronomic and hormonal treatments showed potential for enhancing flowering intensity on sparse and monoecious yam plants with no significant adverse effects on flower fertility and tuber bulking. For better outcomes, yam breeders are advised to use STS when more female flowers are desired, while they should adopt pruning when male flowers are needed to favor the population outcome in a breeding scheme. When facing low fruiting rate, this study recommends using STS and hand pollination to speed up generation of genetically variable offspring for selection, a critical stage in developing and delivering cultivars suiting end-users’ needs and preferences. Such convenient flower induction treatments recommended by this study will be particularly useful to yam breeding programs in developing countries that can hardly afford sophisticated facilities for flower induction.

## Data availability statement

The original contributions presented in the study are included in the article/**Supplementary Material**. Further inquiries can be directed to the corresponding author.

## Author contributions

AA and PA designed the experiment. JM performed data collection. JM and GC performed data analysis. JM drafted the manuscript with inputs from AA and PA. RA, GC, and MA contributed in writing up and revision. MA, PA, and AA performed supervision. All authors contributed to the article and approved the submitted version.
