# Supplementary material for: Agronomic and hormonal approaches for enhancing flowering intensity in white Guinea yam (Dioscorea rotundata Poir.)
Source: Front Plant Sci. 2023 Oct 9;14:1250771. doi: 10.3389/fpls.2023.1250771 (PMC10593412; doi:10.3389/fpls.2023.1250771)
Supplement: Supplementary file 1 [file DataSheet_1.docx]

**Supplementary materials**


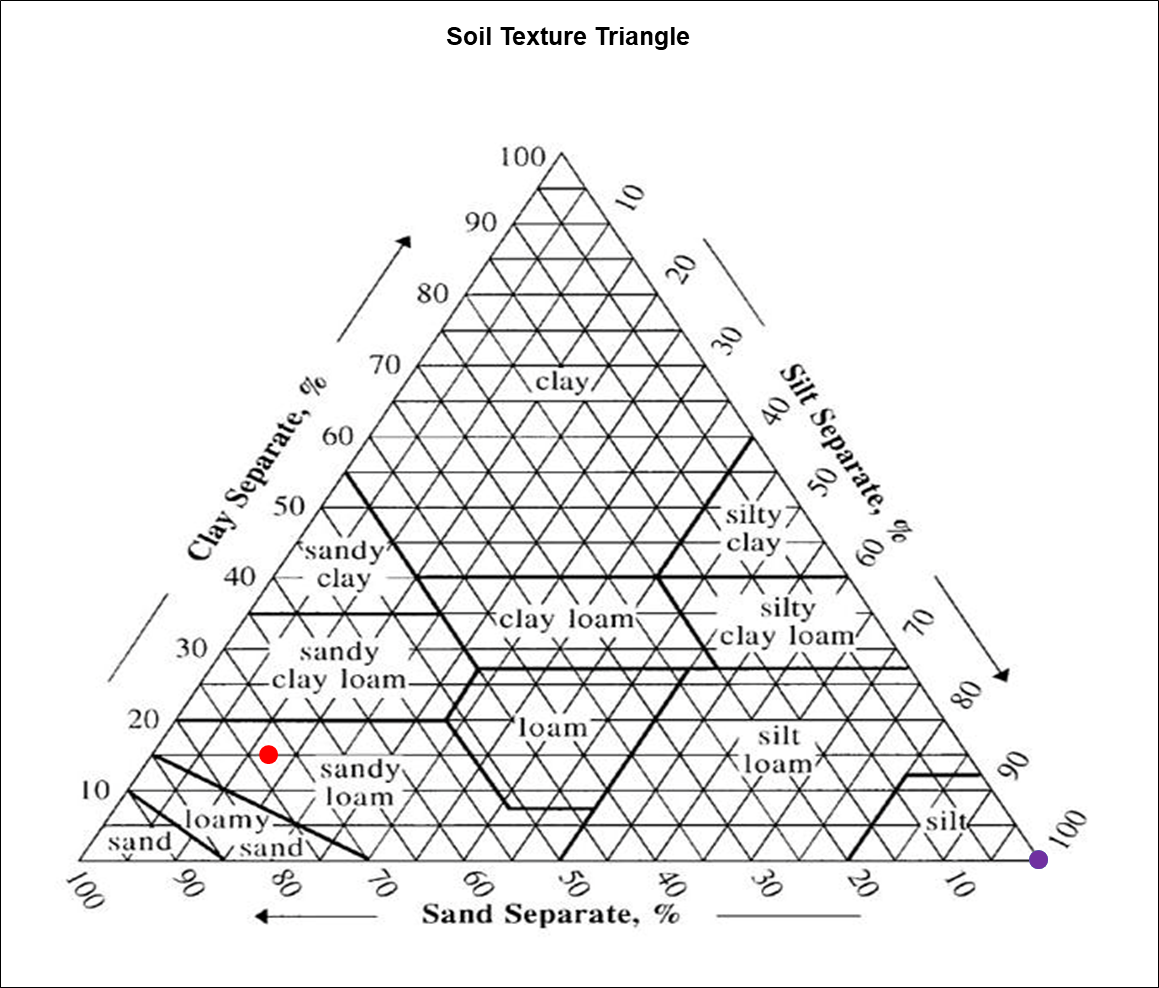


**Figure S1.** Soil texture of the IITA Ibadan station

**Figure S2.** Weekly weather conditions at IITA Ibadan station. Week 1 corresponds to 1^st^ week of July while Week 27 corresponds to the last week of December 2021.


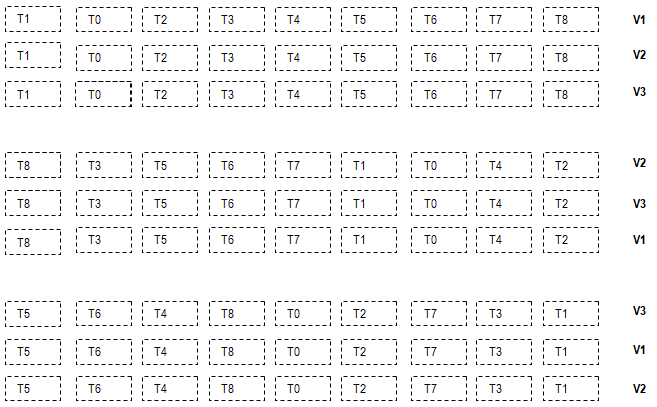


**Figure S3.** Field design showing the arrangement of genotypes and flowering induction treatments.


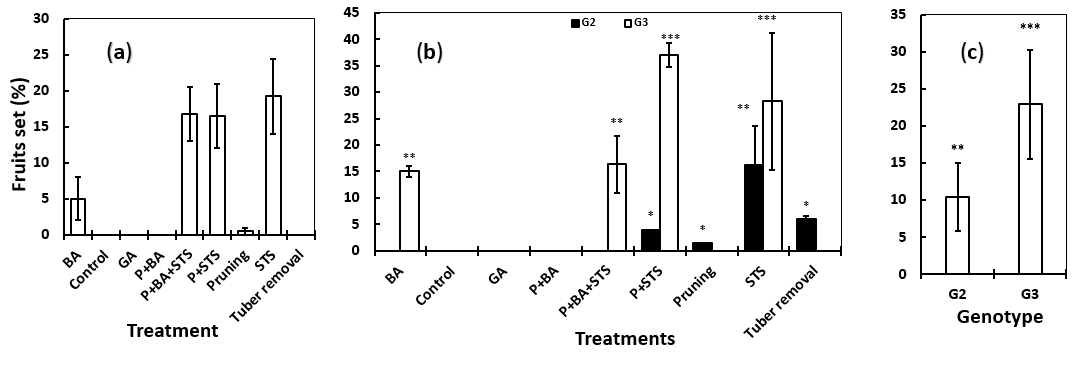


**Figure S4.** Influence of flowering induction treatments on natural pollination in *D. rotundata*: (**a**) average fruit set per flowering manipulation treatment, (**b**) combined effect of the flowering manipulation technique × genotype, (**c**) average fruit set per genotype on fruit set. STS=Silver thiosulfate, BA=Benzyladenine, GA_3_=Gibberelic acid, P=pruning. G2= TDr8902665, G3= TDr1669012.

**Table S1.** Soil characteristics of the study area

| **Parameters** | **Units** | **Values** |
| --- | --- | --- |
| pH(H_2_0) | 1:2.5 | 5.47 |
| OC | % | 0.70 |
| N | % | 0.08 |
| Meh P | ppm | 3.27 |
|  | %Sand | 72.80 |
| Particle size | %Silt | 12.40 |
|  | %Clay | 14.80 |
| Ca | cmol+/kg | 1.42 |
| Mg | cmol+/kg | 0.42 |
| K | cmol+/kg | 0.17 |
| Na | cmol+/kg | 0.08 |
| Exch. Acidity | cmol+/kg | 0.15 |
| ECEC | cmol+/kg | 2.24 |
| Zn | ppm | 29.46 |
| Cu | ppm | 7.36 |
| Mn | ppm | 112.09 |
| Fe | ppm | 54.78 |

OC: organic carbon, Meh P: Mehlich-3 (M3) soil phosphorus, Exch. Acidity: Exchangeable acidity, ECEC: effective cation exchange capacity.

**Table S2.** Summary of data collection

| **Parameters** | **Unit** | **Growth stage** | **Materials** | **Sample size/treatment** | **Variable type** |
| --- | --- | --- | --- | --- | --- |
| Time of flowering initiation | DAP | Flowering | Daily hand count | All plants | Quantitative |
| Number of spikes | - | Flowering | Hand count | All plants | Quantitative |
| Number of flowers/spike | - | Flowering | Hand count | All flowers on 10 spikes | Quantitative |
| Fruit set from hand pollination | % | Flowering | Hand count | All plants | Quantitative |
| Seed set | % | Maturity | Hand count | All plants | Quantitative |
| Number of tubers/plant | - | Maturity | Hand count | All plants | Quantitative |
| Tuber yield/plant | kg | Maturity | Weighing balance | All plants | Quantitative |
| Tuber dry matter content | % | Maturity | Oven-drier | 1 plant per replication | Quantitative |

DAP=Days after planting

**Table S3.** Flowering and fruiting information of *D. rotundata* genotypes

| **Genotypes** | **Flowering (%)** | **Non-flowering (%)** | **Fruiting (%)** | **Non-fruiting (%)** |
| --- | --- | --- | --- | --- |
| Danacha | 0.0 | 100.0 | 0.0 | 100 |
| TDr1669012 | 83.3 | 16.7 | 46.4 | 53.6 |
| TDr8902665 | 28.2 | 71.8 | 10.9 | 89.1 |
| TDr1100875 | 95.2 | 4.8 | 95.2 | 4.8 |
